# Clinical and laboratory postoperative impact on outcome of living donor liver transplantation recipients

**DOI:** 10.1186/cc12410

**Published:** 2013-03-19

**Authors:** Y Nassar, K Omar, A Battah, A Mwafy

**Affiliations:** 1 Cairo University, Giza, Egypt

## Introduction

Adult recipients of living donor liver transplantation have a wide range of postoperative medical comorbidities altering various clinical and laboratory parameters. We aimed to assess 30-day postoperative clinical and laboratory changes and their value in predicting outcome of LDLT recipients.

## Methods

A prospective registry involving patients who underwent LDLTin the period from October 2004 to December 2010. Postoperative 30-day clinical and laboratory data included: clinical BP, HR, temperature, ICU stay, liver functions AST, ALT, GGT, ALP, TotPtn, Alb, TBil, DBil, INR, PTT, CRP,kidney functions Crea, Na, K, blood picture Hb, Hct, TLC and Plt count.

## Results

Our study involved 142 recipients, 124 (87%) males and 18 (13%) females, who underwent living donor liver transplantation; mean age was 53 ± 14.8 years, ranging from 24 to 66 years; 29 cases (20.43%) at Kasr Al Aini University Hospital and 113 cases (79.57%) at the International Medical Center. The 30-day mortality rate was 13.38%. Clinical and laboratory parameters showed gradual resolution towards normal values in survivors or deterioration in nonsurvivors, allowing one to predict mortality at various cutoff points. Clinical parameters: HR >94 cutoff (Acc 76.8%, Sens 89.5%, Spec 62.6%, PPV 27, NPV 97) and ICU stay >8 days (Acc 76.2%, Sens 75.3%, Spec 95.7%, PPV 95.7, NPV 35.3). Laboratory parameters: INR >3.06 cutoff (Acc 87.5%, Sens 92.9%, Spec 78.6%, PPV 96.3, NPV 64.7), followed by PTT >53.3 (Acc 79.2%, Sens 78.6%, Spec 75%, PPV 93.6, NPV 42.9), Na >142.9 (Acc 72.9%, Sens 80%, Spec 72.3%, PPV 26.7, NPV 96.6), TBil >15 (Acc 71.3%, Sens 95.5%, Spec 50%, PPV 92.4, NPV 63.6), ALT >379 (Acc 67.5%, Sens 72.2%, Spec 73.8%, PPV 28.9, NPV 94.7), TProt <4.6 (Acc 63.5%, Sens 55.6%, Spec 73.3%, PPV 24.4, NPV 91.4), AST >145 (Acc 57.4%, Sens 50%, Spec 84.4%, PPV 32.1, NPV 92), Hct <24.4 (Acc 54.2%, Sens 47.4%, Spec 81.5%, PPV 34.6, NPV 88.2), CRP3>12 (Acc 51.6%, Sens 43.7%, Spec 73.3%, PPV 90.5, NPV 17.7), GGT >22 (Acc 51.4%, Sens 83.3%, Spec 36.9%, PPV 16.3, NPV 93.7).

## Conclusion

Predictors of mortality were INR >3.06 (accuracy 87.5%) followed by PTT >53.3 (79.2%), HR >94 (76.8%), ICU stay >8 days (76.2%), Na >142.9 (72.9%), TBil >15 (71.3%), ALT >379 (67.5%), TProt <4.6 (63.5%), AST >145 (57.4%), Hct <24.4 (54.2%), CRP3>12 (Acc 51.6%) and GGT >22 (51.4%).

